# Flexible and Thermally Insulating Porous Materials Utilizing Hollow Double‐Shell Polymer Fibers

**DOI:** 10.1002/advs.202404154

**Published:** 2024-06-25

**Authors:** Joanna Knapczyk‐Korczak, Piotr K. Szewczyk, Krzysztof Berniak, Mateusz M. Marzec, Maksymilian Frąc, Waldemar Pichór, Urszula Stachewicz

**Affiliations:** ^1^ Faculty of Metals Engineering and Industrial Computer Science AGH University of Krakow al. A. Mickiewicza 30 Kraków 30‐059 Poland; ^2^ Academic Centre for Materials and Nanotechnology AGH University of Krakow al. A. Mickiewicza 30 Kraków 30‐059 Poland; ^3^ Faculty of Materials Science and Ceramics AGH University of Krakow al. A. Mickiewicza 30 Kraków 30‐059 Poland

**Keywords:** double‐shell, electrospinning, fibers, mechanical properties, PS, thermal insulation, TPU

## Abstract

The global climate change is mainly caused by carbon dioxide (CO_2_) emissions. To help reduce CO_2_ emissions and conserve thermal energy, sustainable materials based on flexible thermal insulation are developed to minimize heat flux, drawing inspiration from natural systems such as polar bear hairs. The unique structure of hollow double‐shell fibers makes it possible to achieve low thermal conductivity in the material while retaining exceptional elasticity, allowing it to adapt to insulation systems of any shape. The layered system of porous mats reaches a thermal conductivity coefficient of 0.031 W∙m⁻¹∙K⁻¹ and enables to reduce the heat transfer. The results achieved using scanning thermal microscopy (SThM) correlate with the simulated heat flow in the case of individual fibers. This research study brings new insights into the energy efficiency of domestic environments, thereby addressing the growing demand for sustainable and high‐performance insulation materials for saving energy loss and reducing pollution footprint.

## Graphical abstract

Inspired by the insulating properties of polar bear hair, the use of hollow electrospun double‐shell fibers is becoming an excellent option for thermal insulation materials. This innovative approach offers exceptional thermal retention capabilities, demonstrating its potential for applications requiring effective temperature management and energy conservation.

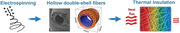

## 1 Introduction

Thermal insulation is crucial in protecting materials exposed to extreme or changing temperatures. Its primary objective is to effectively reduce heat losses associated with heating or cooling, allowing for energy savings.^[^ ^1^, ^2^ ^]^ The decrease in energy consumption contributes to reducing CO_2_ and greenhouse gas emissions.^[^ ^3^ ^]^ The energy conservation of buildings has become one of the most pressing issues at present. The generation of electricity and heating for households is one of the primary contributors to global carbon emissions. Nearly 40% of the world's energy is utilized for heating and cooling buildings.^[^ ^4^ ^]^ Consequently, numerous countries, including EU Member States, have implemented national programs to enhance building energy efficiency.

**Figure 1:**
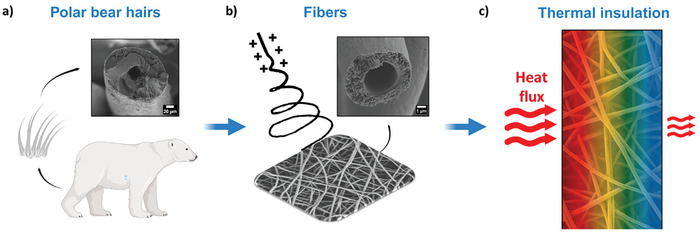
The schematic representation of the concept of this work: a) the biomimetic approach of polar bear hair, b) double‐shell hollow electrospun fibers, c) improving the thermal insulation of obtained fibers via the reduction in heat flux.

Thermal insulation works simply by minimizing heat transfer between two different temperature regions. Insulations with high thermal resistance create a barrier that impedes heat flow, reducing heat flux.^[^ ^5^, ^6^ ^]^ Materials capable of effective heat insulation involve polymer foams,^[^ ^7^, ^8^ ^]^ fiberglass,^[^ ^9^, ^10^ ^]^ mineral wool,^[^ ^11^ ^]^ reflective foils,^[^ ^12^, ^13^ ^]^ aerogels,^[^ ^14^, ^15^, ^16^ ^]^ and natural fibers.^[^ ^17^ ^]^ Thermal conductivity does not depend on the thickness of the insulation, but its thickness primarily influences the thermal resistance. Therefore, increasing the thickness of the material layer reduces energy loss.^[^ ^18^ ^]^ Furthermore, natural materials like cotton and sheep's wool can provide insulation, offering favorable thermal properties. Importantly, many of the recent developments are inspired by nature.^[^ ^19^ ^]^ Polar bears, Tibetan antelopes, and yaks utilize their thick fur, composed of hollow and crimped hairs, to absorb and reflect infrared radiation efficiently, thus effectively maintaining their bodies warm.^[^ ^20^ ^]^ The fur of polar bears (*Ursus maritimus*) possesses unique thermoregulation properties and provides a special adaptation to extremely low temperatures. It consists of protective hair and underfur with diameters ranging from 80–200 and 44–120 µm, respectively.^[^ ^21^ ^]^ They are constructed with an aligned keratin nanofiber cortex containing an inner hollow space called the medulla,^[^ ^22^, ^23^ ^]^ see **Figure** **1**a and Figure S1a,b (Supporting Information). This unique transparent structure reduces heat loss through the scattering and reflection of heat, enabling the possibility of transferring energy from the sun to the animal's body.^[^ ^24^ ^]^ Mimicking the flexible hollow core structure with the air‐trapping and light transmittance properties of polar bear hair can enhance the textile's insulating capabilities, making it a valuable addition to cold‐weather clothing.^[^ ^25^, ^26^ ^]^ Electrospinning is an excellent method to create nature‐inspired fibrous structures that provide adequate insulation in unstable environmental conditions, thanks to increased hydrophobicity that reduces the material's absorbency.^[^ ^27^ ^]^ The geometry of electrospun random fibers provides ≈90% porosity.^[^ ^28^, ^29^, ^30^ ^]^ Via coaxial electrospinning we can produce core–shell fibers,^[^ ^31^ ^]^ by incorporating different polymer.^[^ ^32^ ^]^ The coaxial technique enables the fabrication of hollow fibers as well.^[^ ^33^ ^]^ Notably, the increased void space in fibers reduces the heat‐transport pathways resulting in enhanced thermal insulation.^[^ ^34^, ^35^ ^]^

The primary goal of this research is to create a flexible and thermally insulating porous system with mechanically enhanced hollow fibers, as shown in Figure 1. By biomimicking the structure of polar bear hair via double‐shell hollow electrospun fibers, we want to improve the thermal insulation properties by effectively reducing the heat flux through fibrous mats. We utilized the fiber arrangement in the polystyrene (PS) mat, which exhibits high porosity due to the substantial air content within and between its fibers, resulting in a porous and fluffy appearance driven by charge repulsion effects.^[^ ^36^, ^37^, ^38^ ^]^ However, PS has low mechanical strength, which we improve by incorporating stronger thermoplastic polyurethane (TPU) inside PS fibers. We have successfully verified the correlation between the thermal properties of individual fibers and entire mats. The results show great promise for thermal insulation systems, which can reduce heat losses.

## 2 Results and Discussion

### 2.1 Material Characterization

**Figure 2:**
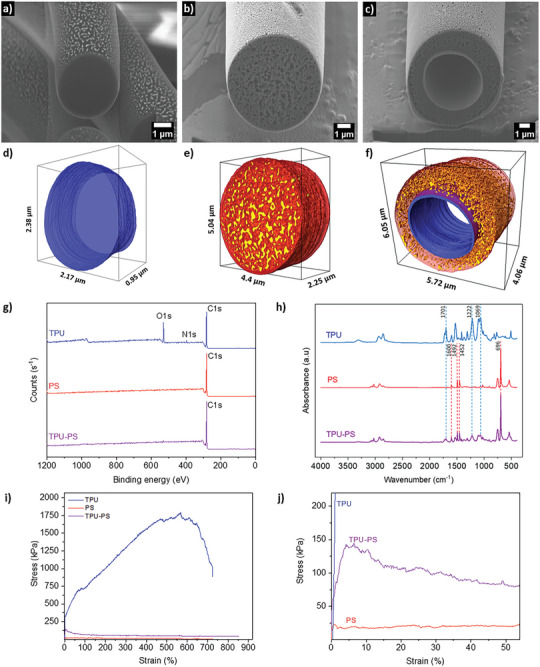
The cross‐sectional views from FIB‐SEM of a) TPU, b) PS, and c) TPU‐PS double‐shell fiber. The 3D tomography of d) TPU, e) PS, f) TPU‐PS. g) XPS spectra. h) FTIR‐ATR spectra. i) Representative stress–strain curves. j) The magnification of stress–strain curves in the deformation range for TPU, PS, and TPU‐PS fiber mat.

We successfully mimicked the structure of polar bear hair, creating TPU‐PS double‐shell fibers with an inner hollow core, as can be observed in Figure 1b and Figure S1h (Supporting Information). The PS and TPU‐PS double‐shell fibers showed similar cotton‐like structure with high average fiber diameters, which reached 4.59 ± 0.36 µm and 5.10 ± 0.53 µm, respectively refer to Figure S1 (Supporting Information). This structure allows us to keep more air in voids between fibers than TPU. The double‐shell fibers have a higher average diameter because of a twice as fast polymer solution flow rate during electrospinning when other process parameters remained the same.^[^ ^39^ ^]^ The primary factors enabling the creation of these double‐shell hollow fibers include solvents, which allow for the rapid and faster evaporation of the shell solution and ensure only partial compatibility between the core and shell solutions.^[^ ^40^, ^41^ ^]^ Moreover, our double‐shell fibers are over ten times smaller than polar bear underfur. The morphology of fibrous mats, histograms with fiber diameter distribution, and the cross‐sectional view of fiber obtained by the freeze‐fracture method are presented in Figure S1c–i (Supporting Information). TPU fibers have almost three times smaller average fiber diameter than PS and TPU‐PS fibers, which reached 1.60 ± 0.15 µm and did not show inner voids. The cross‐sectional views achieved by FIB‐SEM confirmed the presence of pores inside the pristine PS, reaching a value of 23.8 ± 0.9%. Double‐shell fibersinternal porosity was calculated based on the PS shell and spaces inside the TPU core, which gave a value of 37.1 ± 0.9%, see **Figure** **2**a–f and Figure S2 (Supporting Information).

Polar bears must swim and hunt in freezing cold water to survive in the harsh environment.^[^ ^42^, ^43^ ^]^ Therefore, the hydrophobic properties of their fur are crucial because they limit water absorption and thus provide more effective thermal insulation after going ashore.^[^ ^44^ ^]^ Water repellency significantly influences thermal insulation because excessive water absorption can increase the material's thermal conductivity coefficient, which, in turn, results in higher heat flux and greater energy losses.^[^ ^45^, ^46^ ^]^ Therefore, we confirmed the hydrophobic properties of all mats by examining the results of the water contact angle shown in Figure S3 (Supporting Information).^[^ ^47^ ^]^

The XPS results confirm that the surface of the TPU‐PS sample has only PS, and the TPU was not observed, confirming the formation of double‐shelled fibers. Surface concentrations of chemical bonds and analyzed spectra obtained from fitting XPS data^[^ ^48^ ^]^ for all analyzed samples in 8 nm depth are listed in Table S1 (Supporting Information), Figure 2g, and Figure S4 (Supporting Information). The thickness of the PS layer, measured on SEM images, was 1.18 ± 0.44 µm. Therefore, the depth of this analysis did not reach the TPU part.

Further, the chemical composition of electrospun mats was confirmed using Fourier‐transform infrared spectroscopy (FTIR), see Figure 2h with the characteristic spectra of TPU^[^ ^49^, ^50^, ^51^ ^]^ and PS^[^ ^52^ ^]^ in TPU‐PS fibers. These findings confirm the presence of both polymers within the double‐shell fiber mat. A comprehensive study of used polymers and all matching chemical bonds for TPU, PS, and TPU‐PS can be found in Figure S5 and Table S2 (Supporting Information). Additionally, the FTIR analysis of fibers after heating did not reveal any chemical changes in the material, indicating the high thermal stability of the prepared material (Figure S6, Supporting Information). The differential scanning calorimetry (DSC) was used to verify the thermal stability of fibrous mats within the temperature range of 25–100 °C. Notably, the glass transition (T_g_) was detected for PS and TPU‐PS, with respective values of 102 and 103 °C (Figure S7, Supporting Information), closely aligning with the reported T_g_ of 104 °C for PS fibers.^[^ ^53^, ^54^ ^]^ It should be noted that T_g_ was not observed for TPU within this temperature range, and the observed T_g_ for TPU‐PS was related to the T_g_ of PS fibers.

### 2.2 Mechanical Properties

The synergy between TPU and PS enabled the creation of an electrospun double‐shell fiber mat. Surprisingly, even with the presence of the TPU hollow core, this mat exhibited superior mechanical properties compared to a pristine PS mat with fibers of similar diameters, see Figure 2i,j. The TPU mat showed 49 and 13 times higher maximum tensile stress than pristine PS and TPU‐PS double‐shell fibrous mats, see **Table** **1** and Figure S8 (Supporting Information). Implementing TPU inside the PS fiber during coaxial electrospinning results in higher mechanical properties for double‐shell fibers and increases the maximum stress of TPU‐PS up to 4 times compared to a pristine PS mat. This is a combination of tough and flexible TPU with PS, which has low plasticity.^[^ ^54^, ^55^, ^56^ ^]^ The pristine TPU fibers create the highly stretchable mat (Figure S9a–c, Supporting Information), and the plastic deformation can be observed on the individual fiber as a necking effect,^[^ ^57^, ^58^ ^]^ which was opposite to the brittle breakage of PS fiber (Figure S9d–f, Supporting Information).^[^ ^53^, ^56^ ^]^ The breakage of double‐shell fibers was in the PS part, similar to pristine PS, and the TPU was elongated (Figure S9g–i, Supporting Information). The TPU‐PS mat showed the highest maximum elongation at break. However, when the maximum stress was reached, the fibers' strain was still very low, typical of PS. The mat geometry can no longer carry loads after surpassing the maximum stress. Compared to PS, the TPU mat was completely broken along the entire cross‐section at the point of achieving the maximum strain at break. The TPU mat exhibits over 54 times greater toughness than the PS mat. Indeed, integrating TPU into the hollow double‐shell fibers allowed us to achieve a toughness that is 2.5 times higher than what can be achieved with pure PS fibers mat. Apart from the double layer shell, the hollow core and generally the porosity of the mat, play a critical role in increasing the toughness of electrospun mats, including fracture resistance.^[^ ^59^ ^]^ The hollow interior of fibers contributes to stiffness of fibers too.^[^ ^60^ ^]^ However, the increase of porosity of individual fibers, controlled via electrospinning parameters, the maximum stress resistance of mats and fibers can be reduced.^[^ ^61^, ^62^ ^]^ In the case of TPU‐PS fibers, the interfaces between layers enhance the mechanical performance of mats.^[^ ^63^ ^]^ We demonstrated that incorporating flexible and strength TPU into the inner shell of the TPU‐PS fibers improves their strength.

**Table 1:** The mechanical properties obtained from stress‐strain curves.

	TPU	Literature data^[^ ^50^ ^]^	PS	Literature data^[^ ^55^ ^]^	TPU‐PS
Maximum stress (kPa)	1658 ± 208	7800 ± 1500	34 ± 7	5.45 ± 0.86	129 ± 16
Strain at break (%)	720 ± 11	430 ± 140	718 ± 46	29.36 ± 4.14	850 ± 1
Toughness (MJ∙m^−3^)	804 ± 100	‐	15 ± 4	‐	38 ± 10

### 2.3 Thermal Insulation Properties

**Figure 3:**
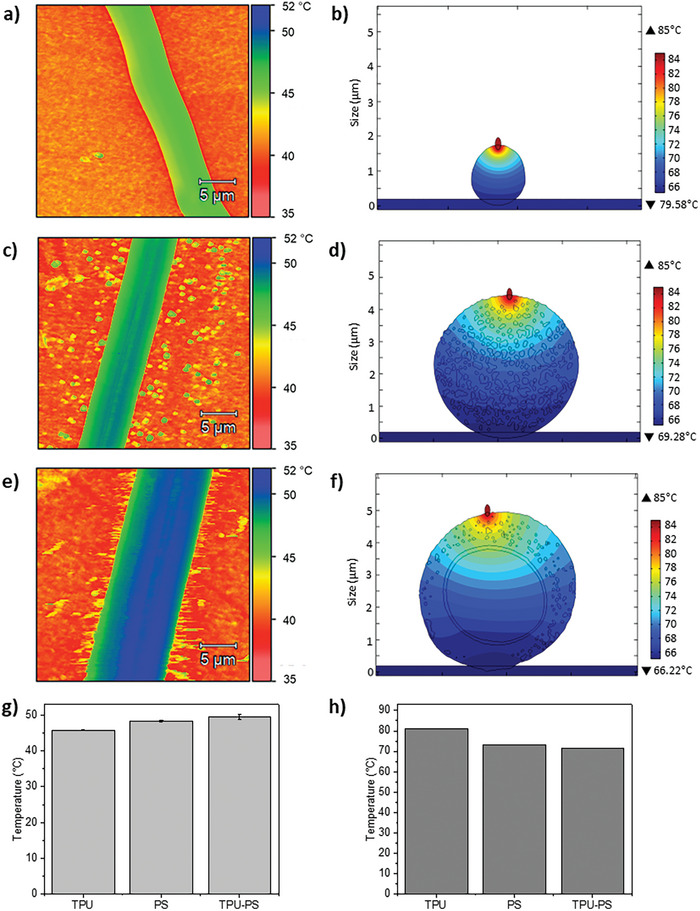
The map of the thermal distribution was obtained via temperature measurements using SThM for a) TPU, c) PS, and e) TPU‐PS fiber. The COMSOL simulation of thermal distribution on b) TPU, d) PS, and f) TPU‐PS fiber. The plotted temperature values obtained from g) SThM measurement and h) COMSOL simulation.

The thermal properties of individual fibers were evaluated using scanning thermal microscopy (SThM), see **Figure** **3**a,c,e,g. The SThM is an effective method for mapping the thermal conductivity of materials, wherein the laser heats the cantilever, and the thermocouple monitors the temperature at its tip. Using SThM, we can investigate thermal conductivity and heat transfer within a single electrospun fiber.^[^ ^64^ ^]^ The heat transfer of an individual TPU and PS fiber and a hollow TPU‐PS double‐shell fiber was monitored by the temperature measurement of the moving SThM tip on the surface of the fibers. The obtained maps display thermal distribution, which is directly connected to the thermal conductivity of different areas on the fiber.^[^ ^64^ ^]^ The heat dissipation between the tip and the sample is enhanced in regions with a higher thermal conductivity, resulting in a reduced temperature at the tip. A higher temperature obtained on fibers indicates higher thermal insulation. Notably, the SThM results for the TPU‐PS fiber demonstrated the highest insulation properties, while the TPU fiber exhibited the lowest. The high porosity of the individual fiber played a significant role, and the TPU‐PS fiber was attributed to the inherent pore space of the PS material and the formation of a void within its core, see micrograph Figure 2c,f, which provided the highest thermal insulation. The hollow fibers' thermal conductivity is lower than the solid fibers. Wang et al. proved that the presence of voids changes the heat transfer mechanism and disrupts the heat flux.^[^ ^65^ ^]^ A gap filled with air, unlike a solid material, exhibits lower thermal conductivity, reducing heat flux. The air‐filled gap impedes heat transfer, primarily traversing through material.^[^ ^40^, ^66^ ^]^ Thanks to this, the hollow structure can break the thermal conduction pathway. Moreover, at the solid‐air interface, the scattering of phonons is observed, which reduces the thermal conductivity of hollow fibers.^[^ ^15^, ^34^ ^]^ Additionally, the heat flux is changed on the material interfaces between the TPU and PS inside the double‐shell fiber because of the thermal conductivity of TPU and PS, which reach respectively 0.23 and 0.18 W∙m^−1^∙K^−1^.^[^ ^67^, ^68^ ^]^

In the simulation results, shown in Figure 3b,d,f,h, we replicated the conditions of the SThM measurement to verify the thermal properties of the obtained materials. The SThM result and simulation agree, showing the same trend for the thermal properties of our samples. The difference in temperature between samples is caused by the way the measurement is carried out in SThM, where the temperature given is measured by the thermocouple located on the apex of the fiber. Thus, higher temperature means higher thermal insulation of the material. In the simulation case, the temperature of the surfaces of fibers is given.

**Figure 4:**
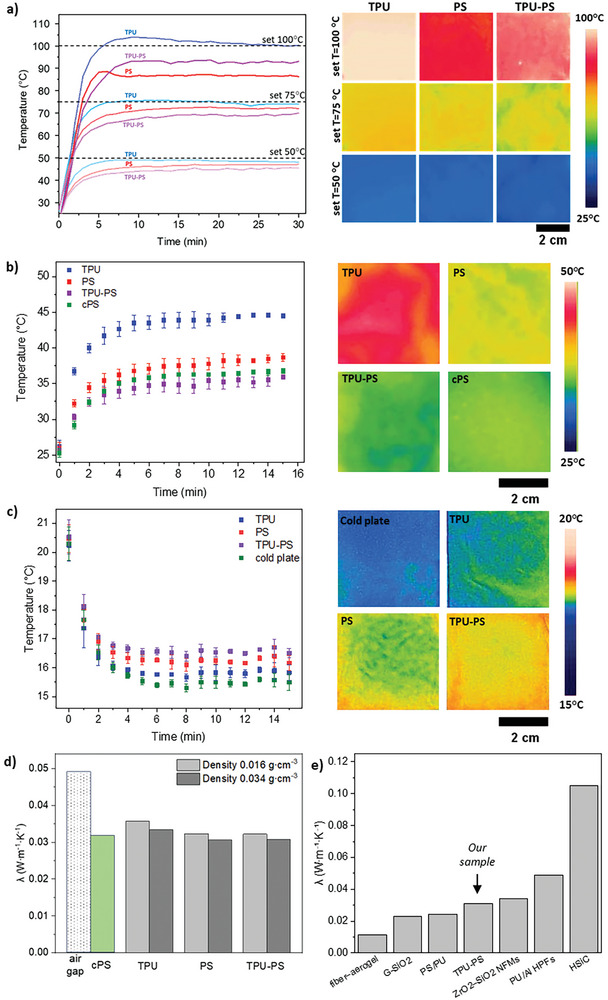
a) The heating curves and thermal images after 30 min of experiment for 1 electrospun layer in three different temperatures set: 50, 75, and 100 °C. b) The heating curves for 4‐layers electrospun mats and cPS in a 50 °C set and their thermal images after 15 min of testing. c) The cooling curves for single electrospun mats and cold plate and their thermal images after 15 min of testing. d) The λ for fibrous samples with density of 0.016 and 0.034 g∙cm^−3^ and for air gap and cPS. Measurement error was <1%. e) The comparison of λ for fiber‐based materials based on publications: An et al.^[^ ^72^ ^]^ (fiber–aerogel), Liu et al.^[^ ^9^ ^]^ (G‐SiO2), Wang et al.^[^ ^65^ ^]^ (PS/PU), Zhang et al.^[^ ^77^ ^]^ (ZrO2–SiO2 NFMs), Hu et al.^[^ ^71^ ^]^ (PU/Al HPFs), Tian et al.^[^ ^78^ ^]^ (HSiC).

Following SThM measurements, an analysis was conducted to assess how the properties of individual fibers correlate with the overall characteristics of the entire mats. The thermal insulating properties of highly porous fiber mats were tested at three distinct temperatures: 50, 75, and 100 °C, see **Figure** **4**a. The mats exhibited closely matched average bulk densities, with PS at 0.014 ± 0.003 g∙cm^−3^ and TPU‐PS at 0.016 ± 0.002 g∙cm^−3^, making them comparable. The average bulk density of TPU reached 0.105 ± 0.021 g∙cm^−3^ because of different geometry and fiber interactions. At temperatures of 50 and 75 °C, the TPU‐PS mat provided the finest heat insulation compared to the pure PS mat because the hollow structure of TPU‐PS fibers reduced the heat flux. The temperature of 100 °C causes the change in the mat's geometry, influencing its thermal insulation properties. The high temperature neutralized the electrostatic charges on fibers, which led to a termination of repulsion between fibers in the PS and TPU‐PS mats.^[^ ^69^, ^70^ ^]^ This effect was not observed previously in lower temperatures. In all three measurements, the geometry of the TPU mat was responsible for showing the lowest insulating barrier. Moreover, the thermal camera test of fibrous mats was performed with 1, 2, 3, and 4 layers at a temperature of 50 °C to verify the influence of thicknesses on thermal insulation performance. The added layers introduce more air pockets to the insulating system. The results showed that by simply increasing the number of layers, we obtain a higher reduction in heat flux, thus increasing the thermal insulation, see Figure S10 (Supporting Information). The highest decrease in the temperature on the sample surface was obtained in a four‐layer system. Both thermal camera measurements were conducted for comparison using commercial extruded PS (cPS), see Figure S11 (Supporting Information). We achieve improved insulating properties by using just four layers of porous TPU‐PS mats (≈4.4 mm in thickness) with the half bulk density of cPS (Figure 4b). It should be noted that both materials differ in spatial geometry and pore type. The experiment was supplemented with an investigation into the insulation properties of the fibers when in contact with a colder surface. The study, conducted on a single layer of fibers mat (Figure 4c), clearly demonstrates that double‐shell hollow fibers exhibited superior insulation performance compared to pure TPU and PS mats. The temperature difference observed between the TPU‐PS mat and the colder surface was 1 °C, whereas for TPU and PS, this value was 66% and 30% lower, respectively.

However, the flexibility of the fibrous mats increases their range of applications due to the lack of shape limitations. Hu et al. proposed elastic hollow porous TPU fibers with reflective thin Al platelet skin, inspired by sunlight shielding Saharan silver ant and polar bear hairs, obtained via a coaxial wet spinning method.^[^ ^71^ ^]^ The obtained textile was characterized by low thermal conductivity, reaching 0.049 W∙m^−1^∙K^−1^ and IR emissivity of ≈0.36. Low IR emissivity was due to the Al layer, which protects the material against excess heat transported by thermal radiation. An et al. proposed a flexible, 3D network with hydrophobic and thermal‐insulating properties.^[^ ^72^ ^]^ The composite membranes consisted of ceramic‐based fibers and silica aerogels and were characterized by a low thermal conductivity of 11.4 mW∙m^−1^∙K^−1^. Furthermore, they confirmed that, in the case of porous fibrous materials, a slight change in material thickness enhances its insulating properties. In general, thickness is a crucial parameter for a thermal insulation layer because it affects the length of the heat flow pathway and the heat flux value, which correlates with Fourier's law of heat conduction.^[^ ^73^ ^]^ Omitting the thickness information of tested samples in scientific works eliminates the possibility of comparing the insulation performance of materials.

Fibrous insulation materials prevent heat transfer by reducing conduction by incorporating materials with low thermal conductivity. They also decrease convective heat transfer by trapping air within the material. The third mechanism of heat transfer in high‐porosity fibrous materials is radiation, which rises with increasing heating temperature but can be controlled by reflective surfaces.^[^ ^74^, ^75^ ^]^ Zhou et al. proposed a coaxial fibers system with a cellulose nanofibril aerogel core and a cellulose‐rich sheath.^[^ ^76^ ^]^ The unique combination of a porous sheath and an aerogel core minimized heat conductivity through all three mechanisms. The air circulation limited convective heat transfer, while the poorly conducting cellulose decreased conductive heat transfer. Moreover, the aerogel cellular walls were created in this system to reduce infrared radiation.

Our research clearly demonstrates a correlation between the insulating properties of individual fibers and entire mats. In both scenarios, the most significant reduction in heat flux was observed with hollow double‐shell TPU‐PS, exhibiting the highest thermal resistance. The observations made at the macroscale for mats show the same trend as previously observed for single fibers.

The thermal conductivity is determined by evaluating the ratio of the measured heat flux to the temperature difference across the thickness of the sample.^[^ ^74^ ^]^ Importantly, to design the hollow double‐shell fibers system, we used low thermal conductivity materials, which significantly reduced the heat transfer through the fibers.^[^ ^79^, ^80^ ^]^ Within our study, we measured the thermal conductivity coefficients (λ) of electrospun highly porous mats using the standard heat flow meter.^[^ ^81^, ^82^ ^]^ Tests were conducted for two bulk densities, 0.016 and 0.034 g∙cm^3,^ and for the lower value, obtaining 0.036 W∙m^−1^∙K^−1^ for TPU and 0.032 W∙m^−1^∙K^−1^ for PS and TPU‐PS mats, see Figure 4d. The combination of TPU with PS in double‐shell fibers resulted in a similar λ as pristine PS fiber. However, TPU in the fiber core contributed to an increase in the mechanical strength of the TPU‐PS fiber mat. The higher density of samples 0.034 g∙cm^3^, which is close to the density of the used cPS, was obtained by applying a higher compression to the mats, lowering the λ to 0.033 W∙m^−1^∙K^−1^ for TPU and 0.031 W∙m^−1^∙K^−1^ for PS and TPU‐PS, respectively. For the commercial material, it was 0.032 W∙m^−1^∙K^−1^. Our fibrous mats have similar insulation properties but can be adapted to any surface. They are flexible, opening enormous application opportunities. In our system, heat transfer occurred partially via conduction within the hollow double‐shell fibers,^[^ ^83^, ^84^, ^85^ ^]^ and convection because of high porosity and opened pores in materials. The measurements show that the presence of mats with hollow double‐shell fibers in the air gap decreased the λ due to a change in the heat transfer mechanism and disruption of the convection. The air gap indicated >30% higher values of the λ, indicating excellent improvement of thermal insulation just by using our bioinspired double‐shell fibers in the randomly oriented mat. A comparison of the λ obtained for our double‐shell hollow fibers with the literature values for other fiber‐based materials discussed in this manuscript is shown in Figure 4e.

**Figure 5:**
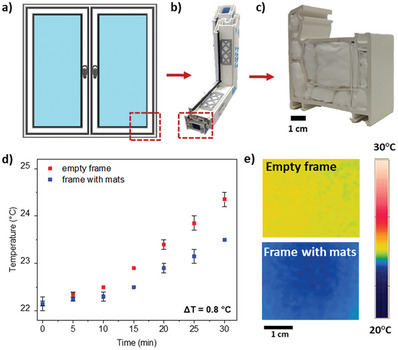
a) The scheme of a window, b) the cross‐sectional view of a window frame, and c) TPU‐PS fibers inserted inside the window frame. d) The heating curves illustrate the impact of fibers on the thermal insulation of the window frame. e) The thermal image after 30 min of the experiment of the measured surface of the window empty frame and frame with fibers inside.

To verify the application of hollow double‐shell fibrous mats, we consider the most straightforward challenge, which is heat loss occurring in households through windows. One effective strategy to mitigate energy losses involves reducing frame conductance, for instance, by insulating the window frames.^[^ ^86^, ^87^ ^]^ The mats with hollow double‐shell fibers were used as insulation material for the window frame, as shown in **Figure** **5**a–c. A temperature change was observed on the sample's surface after 30 min of heat flow through the frame, giving values of 1.4 ± 0.1 and 2.2 ± 0.1 °C, respectively, for the filled and unfilled fiber profiles, as seen in Figure 5d,e. The use of fibers inside the window frame profile decreased the heat flow, resulting in a reduction in surface temperature by 0.8 °C. Reducing heat loss through window frames refers to decreasing the temperature difference between the inside and outside of the window frame. The impact of this reduction can vary depending on factors such as the size of the window, the type of frame material, and the climate conditions. Even a small reduction in heat loss can lead to energy savings, as it means less energy is required to maintain the desired indoor temperature.^[^ ^88^ ^]^ This can translate into lower heating costs during colder months. The reduction of heat flow by simply changing the insulation level of the window plays a crucial role in reducing the heating loss and the upkeep costs of the whole house.^[^ ^89^ ^]^ By reducing the energy needed for heating, there is a corresponding reduction in greenhouse gas emissions associated with energy production.^[^ ^90^ ^]^ This contributes to mitigating climate change and reducing the overall environmental footprint.

## 3 Conclusion

We have replicated the unique properties of natural hollow polar bear hair to create thermally efficient, flexible, and lightweight mats based on hollow double‐shell fibers that can be used for basic thermal insulation in houses and beyond. To create hollow double‐shell fibers, we introduced TPU inside, which improved the mechanical properties of the resulting TPU‐PS fiber. Thanks to the low thermal conductivity of PS and the hollow space inside the double‐shell fibers, heat transfer was reduced both for the individual fiber and for the layered system of mats. The effective thermal insulation of our biomimetic system was confirmed for mats with the thermal tests and measurement of heat transfer through individual fibers via SThM. The theoretical analysis of heat transfer through hollow double‐shell fibers confirmed the reduced heat flux in our polymer fiber systems compared to pure TPU and PS. This innovative approach paves the way for improved thermal insulation of fibrous materials and highlights the potential for mimicking nature's solutions in materials engineering. Improving the energy efficiency of buildings has the potential to make a significant contribution to reducing CO_2_ emissions and the overall carbon footprint.

## 4 Experimental Section

### Sample Preparation and Characterization

*Materials and electrospinning*: Thermoplastic polyurethane (TPU, Elastollan 1185 A15, BASF, Germany) and polystyrene (PS, product no. 441147‐1KG, M_w_ ≈350 000 g∙mol^−1^; Sigma–Aldrich, USA) were dried at T = 30°C (laboratory drying machine, Pol‐Eko, Poland) until the moisture evaporated completely and then dissolved in dimethylformamide and tetrahydrofuran solvents (DMF, (ACS) pure P.A.; THF, (ACS) pure P.A., Avantor, Poland). Solutions of PS 25%_ wt._ in DMF and TPU 15% _wt._ in THF‐DMF with a 1:1 volume ratio were stirred at a constant speed of 400 rpm for 4 h at T = 25 °C (RCT basic, IKA, Germany). Fibers were produced by electrospinning (NanoFiber Electrospinning – ESVY‐100, MicroNano Tools, Canada) using a standard stainless‐steel nozzle (OD = 0.8 mm, gauge G21, length = 4 cm) to prepare PS and TPU mats and a coaxial nozzle (for core ID = 0.3 mm and shell ID = 1.3 mm) to obtain TPU‐PS fibers with TPU core inside and PS shell outside, see Figure S12 (Supporting Information). The electrospinning was performed in the environmental control chamber with the parameters listed in **Table** **2**. The samples were electrospun on the rotating collector drum (10 rpm) onto Al foil for the characterization and the thermal measurements (TPU and PS for 2 h, TPU‐PS for 1 h) and onto paper frames for the tensile test for 30 min. A short electrospinning process (10 s) was carried out to prepare samples on the indium tin oxide‐coated glass slide (ITO glass, Ossila, UK) for the scanning thermal microscopy (SThM) tests. The ITO glass was used as a substrate to maintain proper adhesion between fibers and the background surface to obtain a high‐resolution thermal map.

**Table 2:** Electrospinning parameters for all the produced samples.

*Electrospinning parameters*			
	TPU	PS	TPU‐PS
Flow rate [mL·h^−1^]	1.5	1.5	TPU core = 1.8 PS shell = 3.6
Distance [cm]		20	
Applied voltage [kV]	13	11	26
Temperature [°C]	25	25	28
Relative humidity [%]	40	40	40

*Scanning Electron Microscopy*: Fibers were analyzed by a scanning electron microscope (SEM, Merlin Gemini II, ZEISS, Germany). All samples were coated with an 8 nm Au layer in a rotary pump sputter coater (Q150RS, Quorum Technologies, UK). The accelerating voltage, current, and working distance used were 2.5 kV, 110 pA, and 6 mm, respectively. The average fiber diameters were determined using ImageJ software (version 1.50i, National Institutes of Health, USA) and calculated from 100 measurements for each sample. The PS shell in hollow double‐shell TPU‐PS fibers was derived from 50 measurements. Histograms of fiber size distribution were prepared using the OriginPro software (2018b, OriginLab, USA). Moreover, the SEM was used to measure the thickness of mats for mechanical testing on vertically placed samples using the ImageJ software and to check the morphology of fibers after tensile tests.

The freeze fraction method was used to obtain the cross‐section view of fibers. In this case, the fibrous mats with random orientation were put into liquid nitrogen to freeze and then were cut with a scalpel. SEM imaging was done for vertically oriented fibers placed on the Al stub. The same procedure was used to prepare SEM images of the cross‐sectional view of polar bear hairs.

*3D Reconstruction with Focused Ion Beam Tomography*: 3D reconstructions of fibers were prepared using a focused ion beam scanning electron microscope (FIB‐SEM Crossbeam 350, ZEISS, Germany). Before imaging, the fibers were put on an Al stub and coated with a 15 nm layer of Au. The SEM imaging was done with an accelerating voltage of 2.5 kV and a working distance of 3 mm. The voltage used for FIB tomography was 30 kV, and the current was 20 pA for TPU and 50 pA for PS and TPU‐PS fibers, respectively. The Avizo software (ThermoFisher Scientific) was used to prepare 3D tomographies. The porosity of a single fiber was calculated using the “Analyze Particle” option in ImageJ software on 20 randomly chosen images, followed by the standard deviation calculation. The porosity for the double‐shell fiber was calculated based on the pore area of the PS shell and the hollow space inside the fiber core.

*Wetting*: The wetting properties were checked in the static contact angle measurement. Deionized water droplets (DI, Spring 5UV purification system – Hydrolab, Poland) with a volume of 3 µL were applied on horizontally placed fiber samples at environmental conditions of T = 22 °C and RH = 30%. Images were taken 5 s after the deposition of droplets on the surface by the camera with a macro lens (EOS 700D, EF‐S 60 mm f/2.8 Macro USM, Canon, Japan). The static contact angle was determined by analyzing images of ten different droplets using ImageJ software, and the standard deviation was calculated.

*X‐Ray Photoelectron Spectroscopy*: The X‐ray photoelectron spectroscopy (XPS) analyses were carried out in a PHI VersaProbeII Scanning XPS system using monochromatic Al Kα (1486.6 eV) X‐rays focused to a 100 µm spot. The photoelectron take‐off angle was 90°, and the pass energy in the analyzer was set to 46.95 eV (0.1 eV step) to obtain high energy resolution spectra for the C 1s, N 1s, and O 1s regions. A dual beam charge compensation with 7 eV Ar+ ions and 1 eV electrons was used to maintain a constant sample surface potential regardless of the sample conductivity. All XPS spectra were charge referenced to the unfunctionalized, saturated carbon (C‐C) C 1s peak at 285.0 eV. The operating pressure in the analytical chamber was <2 × 10‐9 mbar. Deconvolution of spectra was carried out using PHI MultiPak software (v.9.9.3). Spectrum background was subtracted using the Shirley method.

*Fourier Transform Infrared Spectroscopy*: The Fourier transform infrared spectroscopy (FTIR) was used to analyze the molecular structure of obtained TPU and PS fibers and to confirm the contents of both polymers in the double‐shell fibers (Nicolet iS5, Thermo Fisher Scientific, USA). The analysis was performed with a resolution of 4 cm^−1^ and 400–4000 cm^−1^ range. For each sample, 64 scans were performed using the diamond ATR module (iD7 ATR – Diamond). The data was analyzed using OMNIC 9 software (version 9.12.928, Thermo Fisher Scientific, USA) and the OriginPro software (2018b, OriginLab, USA) to prepare graphs.

*Differential Scanning Calorimetry*: As previously reported, thermal analysis was conducted with differential scanning calorimetry (DSC, Thermal Analysis System DSC 3, Mettler Toledo, Switzerland) in the temperature range from 25 to 200 °C at a heating rate of 10 K∙min^−1^.^[^ ^53^ ^]^ These studies have established a maximum temperature limit of 100 °C for thermal measurements using a heating plate.

### Mechanical Properties

The mechanical properties of the fibrous mats were verified using the tensile module (Kammrath Weiss GmbH, Germany) equipped with a 20 N load cell. For this purpose, fibers were put on paper frames with a gap of 1.8 × 2 mm and mounted between clamps. Afterward, the paper was cut, and samples were uniaxially stretched with an extension rate of 25 µm·s^−1^. The test was performed three times for each sample type, including TPU, PS, and TPU‐PS double‐shell fibers. Stress–strain curves, prepared with OriginPro software, were used to calculate the maximum stress, strain at break, and toughness (Origin integrate function). Obtained average values were calculated from three tests with a standard deviation.

### Thermal Insulation Properties

*Scanning Thermal Microscopy (SThM)*: The thermal analysis on the individual fiber was performed using an atomic force microscope (AFM, CoreAFM, Nanosurf, Switzerland) with a specialized scanning thermal microscopy module (SThM, VertiSense, AppNano, USA), see Figure S13 (Supporting Information). A VTP‐200 thermal probe (manufactured by AppNano, USA) was utilized to conduct the thermal measurements. The probe featured a Si cantilever with a spring constant of 9.9 N.m^−1^ and a hollow silicon dioxide tip, which integrated a 50 nm thermocouple at the tip's apex. The cantilever tip was heated using a laser, and its temperature was precisely maintained at 60 °C above room temperature (24 °C) before it made contact with the sample. The ITO substrates were securely attached to a metallic stage using a silver paste, ensuring an effective thermal path for heat dissipation. During the measurements, the AFM was set to operate in topography mode with a scanning rate of 2 s per line and a resolution of 256 pixels per line. This configuration allowed sufficient time for heat transfer to take place. The same thermal probe was used for all measurements to eliminate any potential differences in thermal transport that could arise from using different probes. Furthermore, all samples were simultaneously affixed to the identical metallic stage for the same purpose. All measurements were performed at T = 24 °C and RH = 55%. Three measurements for each sample type were conducted at three randomly selected locations. The result for the ITO sample represents a composite of backgrounds from all the measurements that were taken. The ITO was tested along the fibers to serve as a reference to ensure that measurement was not affected by external factors such as room temperature fluctuations, substrate variation, or thermal conductivity issues.

*Computational Simulation*: The simulation of heat transfer in the fibers was performed by COMSOL Multiphysics (version 5.6, COMSOL Inc., Sweden). Due to the high length‐to‐diameter ratio of the yarns used in measurements, the temperature was assumed to change only in the radial direction. Thus, fibers were simplified to 2D models that were reproduced from slices obtained with FIB‐SEM tomography. Convective heat flux for external natural convection of long horizontal cylinder setting was set for simulation. The diameters of fibers used in the simulation were set to the average value calculated from SEM images for all fibers: 5.1, 4.59, and 1.6 µm for TPU‐PS, PS, and TPU, respectively. The TPU and PS thermal conductivity were set to 0.32 and 0.189 W∙m^−1^∙K^−1^, respectively. The voids were assigned the value for Air 0.021 W∙m^−1^∙K^−1^. The fibers were placed on the plate resembling ITO Glass, which was assigned a thermal conductivity of 10.2 W∙m^−1^∙K^−1^. The heat transfer coefficient for fibers was set individually based on their diameter using external natural convection for long horizontal cylinders. The thermal conductivity of ITO support was set to 10.2 W∙m^−1^∙K^−1^, based on the literature values.^[^ ^91^ ^]^ Ellipsoids representing AFM tip apex used as heat sources were set to 85 °C to resemble the conditions in the SThM measurement. The ambient temperature was set to 25 °C. The simulation results presented in Figure 3 were performed at the stationary conditions after reaching the temperature equilibrium. The temperature of fibers compared to an actual temperature reading from SThM results was calculated using the surface average function in the COMSOL Multiphysics software.

*Thermal Camera Measurement*: Thermal insulation properties were verified using the heating plate (TLC plate heater III, CAMAG, Switzerland) and thermal camera measurement (T560, FLIR, USA), see Figure S14a,b (Supporting Information). Samples with dimensions 4 × 4 cm were horizontally placed on the heating plate starting at T = 25 °C and RH = 30‐35%. The sample emissivity was calculated using the vinyl electrical tape (3m Super 33+, Scotch) with the known emissivity, reached 0.96 and thickness of 150 µm. The temperature comparison between the heating plate and the 3m tape yielded comparable results. The distance between the thermal camera and the heating plate was 70 cm. Samples were placed horizontally on the heating plate and then heated from environment temperature to a set temperature for 30 min. Temperature was set to 50, 75, and 100 °C. Thermal images were taken every 1 min to observe temperature changes at the top of the layers. The initial 10 min of measurement represented the time during which the heating plate heated up and stabilized. After this period, equilibrium could be observed on the heating curves. Thermal insulation properties were checked using 1 to 4 number of layers of all mats. The fiber morphology and their molecular structure after thermal measurement on the heating plate at 100 °C were checked by SEM imaging and FTIR analysis. The thermal camera measurement was repeated three times at 50 °C, and the heating time was reduced to 15 min. Previous tests have shown that this time is sufficient to achieve stable temperature values on heating curves.

Thermal camera measurements verified the insulation properties of single‐layer mats in contact with the cold plate. Similar to a previous study, samples with dimensions of 4 × 4 cm and an initial temperature of 20 °C were horizontally placed on a 5 mm expanded polystyrene plate (EPS), which was then put on ice packs with temperatures below 0 °C. The ice packs were placed in a specially designed styrofoam thermal box before the test. The EPS plate allowed for uniform temperature distribution during measurement and prevented moisture condensation on the tested fibers, which could affect the results and increase thermal conductivity. Changes in the temperature of the EPS plate and fiber mats were recorded with a thermal camera for 15 min after being placed on the ice. See the schematics of the experimental setup in Figure S14c (Supporting Information).

The thickness of samples was controlled by flow rate and electrospinning time for fiber production and the number of electrospun mats, which ranged from 1 to 4 layers. For this purpose, it was decided to place the individual mat systems between the glass slide and cover glass on top of fibers and measure the thickness via confocal laser scanning microscope (Zeiss LSM 900 confocal microscope, Zeiss, Germany) from the difference of Z position located on the cover glass on the top of fibers and the top of the glass slide, see Figure S15 (Supporting Information). Thickness was measured for every sample in five different places on the cover glass because of the rough surface of the mat. The thicknesses of the prepared layer systems are added to Table S3 (Supporting Information). All samples were weighed on the analytical balance (XPR 105 Delta Range, Mettler Toledo, Switzerland). Then, the received weight was divided by the calculated volume from the sample's thickness and surface area to obtain a bulk density of electrospun mats, see Table S4 (Supporting Information).

*Thermal Conductivity Coefficient Measurement*: The thermal conductivity coefficient (λ) was measured using a FOX 50 heat flow meter (Laser Comp, USA) calibrated on the Pyrex glass standard. The software calculated the λ according to Fourier's Law and based on average heat flux. The value of the λ was calculated from 256 counts from the block, which had a stable heat flux value. All tests were performed with the temperature gradient of 5 °C in the system, where the top plate was hotter than the down plate, which prevented the natural convection from influencing the results, see Figure S16 (Supporting Information). The presented results were calculated from the last three stable blocks, where the measurement error was <1%. In this test, fibers were put into a measuring gap with a diameter of 60 mm and a height of 5 mm between two plates. The temperature of the top and down plates was measured by the set of thermocouples with a measuring surface with a diameter of 25 mm at the center of the plate. The mass of fibers in every measuring gap was the same, which allowed to obtain a similar bulk density for each material. Bulk density was calculated as a mass‐to‐volume ratio of the sample. The experiment was performed for two bulk densities of 0.016 and 0.034 g∙cm^−2^. The lower bulk density value was close to PS and TPU‐PS values in the thermal camera measurement and was obtained by filling the entire height of the measuring gap. The higher bulk density was similar to the bulk density of compared commercial extruded PS and required compressing fiber mats. Moreover, doubling the density of fibrous samples was allowed to verify this effect on insulating properties. The TPU sample was prepared in a way that allows it to achieve the same bulk density as for the pristine PS and TPU‐PS. The single TPU mat was thin, but the layered structure of the sample guaranteed the presence of an air volume arranged between its layers. For PS and TPU‐PS, a lot of air was retained in the gaps between the fibers, which repelled each other due to their electrostatic charges’ interaction.^[^ ^92^ ^]^ To compare the results, we used commercial extruded polystyrene insulation materials (cPS, Hoch, Poland). The bulk density and λ reached 0.033 g∙cm^−2^ and 0.032 W∙m^−1^ K^−1^, respectively. The thickness of the samples was 2 and 5 mm for the thermal camera and λ measurements.

*Verification of the Material's Applicability*: The thermal camera measurements were also conducted on a sample extracted from the window frame (Oknoplast, Poland) with dimensions of 7 cm in height and 3 cm in width. The heating plate was set to a temperature of 50 °C, and the distance to the thermal camera was maintained at 70 cm (see Figure S17, Supporting Information). The experimental setup involved two scenarios: one with the frame devoid of fibers and another with the frame filled with TPU‐PS fibers weighing 1.76 ± 0.0001 g. To ensure uniform insulation, 2 cm of cPS surrounded the frame on all four sides. Every measurement was conducted for 30 min with two repetitions.

## Conflict of Interest

The authors declare no conflict of interest.

## Supporting information

Supporting Information

## Data Availability

The data that support the findings of this study are available from the corresponding author upon reasonable request.
