# Supplementary material for: Flexible and Thermally Insulating Porous Materials Utilizing Hollow Double‐Shell Polymer Fibers
Source: Adv Sci (Weinh). 2024 Jun 25;11(36):2404154. doi: 10.1002/advs.202404154 (PMC11423226; doi:10.1002/advs.202404154)
Supplement: Supplementary file 1 — Supporting Information [file ADVS-11-2404154-s001.docx]

**Supporting Information to**

Flexible and thermally insulating porous materials utilizing hollow double-shell polymer fibers

*Joanna Knapczyk-Korczak^1^, Piotr K. Szewczyk^1^, Krzysztof Berniak^1^, Mateusz M. Marzec^2^, Maksymilian Frąc^3^, Waldemar Pichór^3^, and Urszula Stachewicz^1*^*

^1^Faculty of Metals Engineering and Industrial Computer Science, AGH University of Krakow, al. A. Mickiewicza 30, 30-059 Kraków, Poland

^2^Academic Centre for Materials and Nanotechnology, AGH University of Krakow, al. A. Mickiewicza 30, 30-059 Kraków, Poland

^3^Faculty of Materials Science and Ceramics, AGH University of Krakow, al. A. Mickiewicza 30, 30-059 Kraków, Poland

*Corresponding author: Urszula Stachewicz

E-mail: ustachew@agh.edu.pl; Tel.: +48 12 617 52 30

**Keywords:** electrospinning, fibers, double-shell, thermal insulation, mechanical properties, PS, TPU


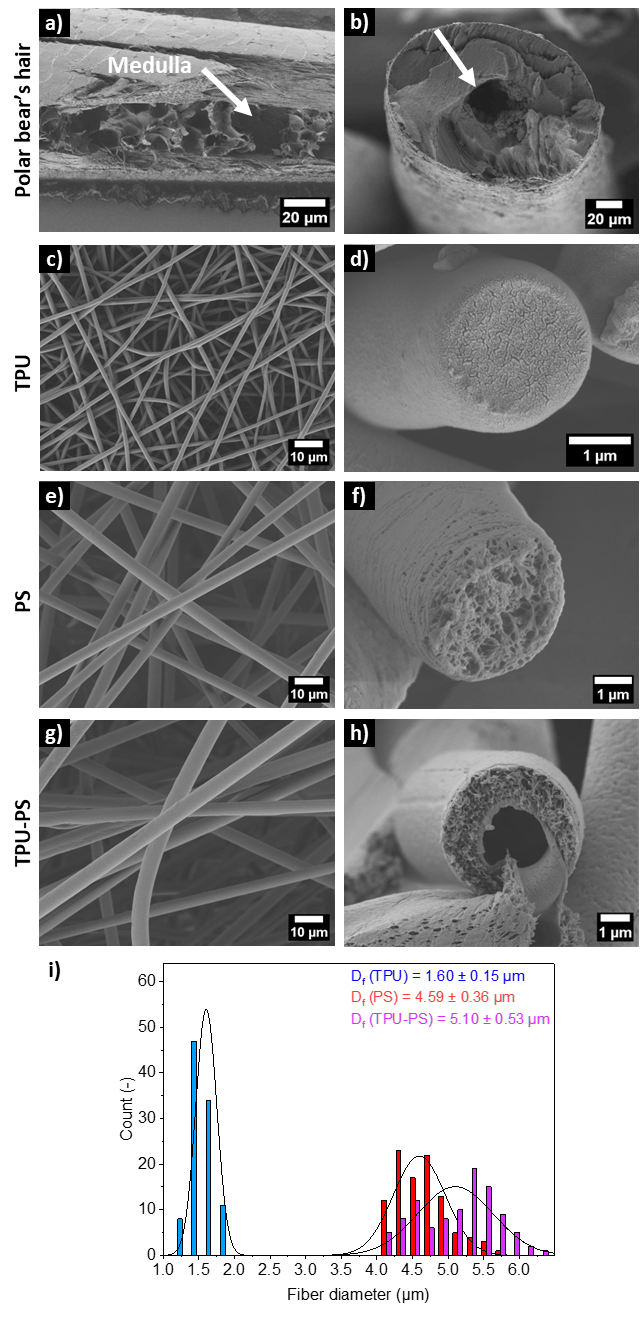


**Figure S1.** The cross-sectional views of the empty medulla of a polar bear’s hair: a) along and b) across the hair. The top view of c) TPU, e) PS, and g) TPU-PS fibers. The cross-sectional views from freeze-fracture of d) TPU, f) PS, and h) TPU-PS fiber. i) The fiber diameter distribution.

**
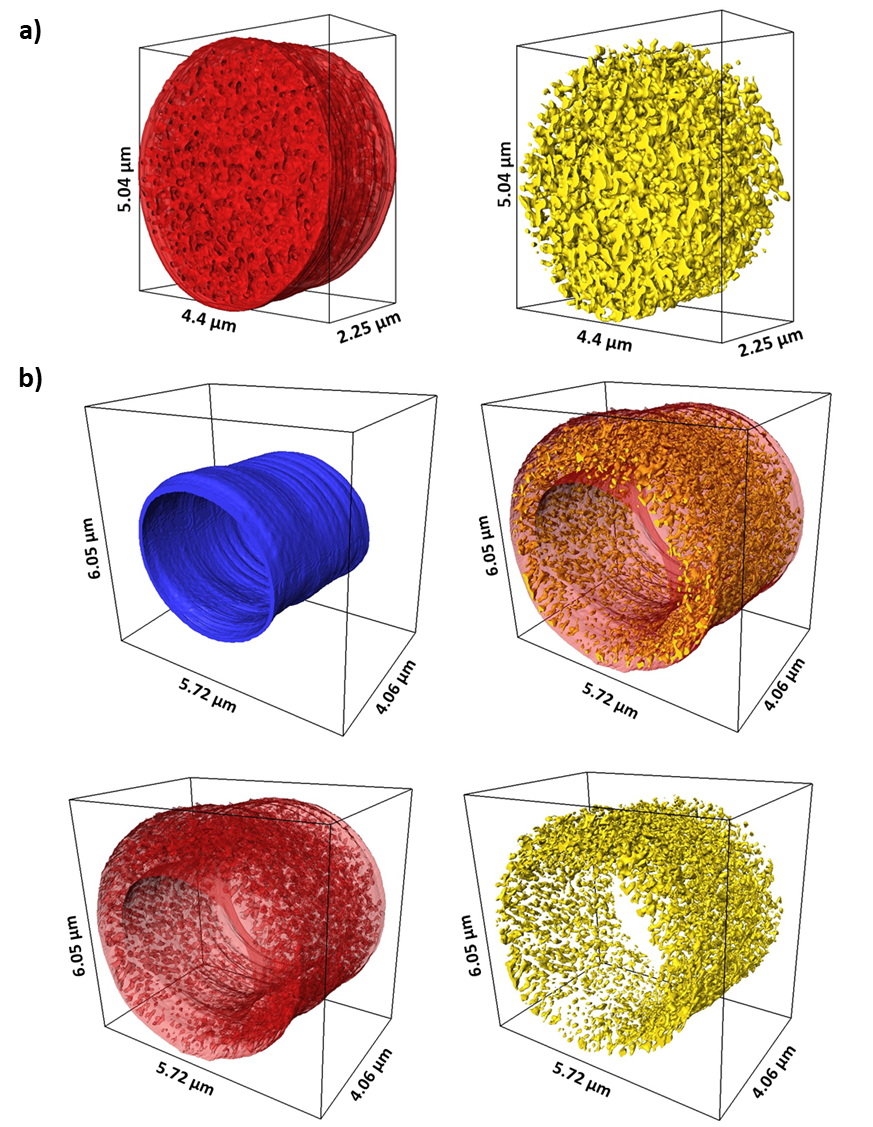
**

**Figure S2.** The reconstruction of FIB-SEM tomography for a) PS fiber, b) TPU-PS divided to TPU core and PS shell. Blue color is for TPU, red is for the PS and yellow is for pores.

In our study, we investigated the wetting properties of prepared mats of TPU, PS and TPU-PS. All mats exhibited a water contact angle greater than 90°, indicating a significant resistance to water infiltration and an increased hydrophobicity. The obtained values for TPU, PS, and TPU-PS reach 125 ± 4°, 136 ± 6° and 139 ± 8°, respectively.


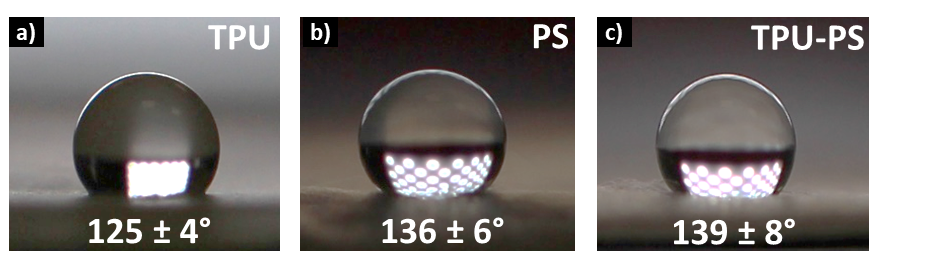


**Figure S3.** The droplets on hydrophobic fibers: a) TPU, b) PS, and c) TPU-PS.

The C 1s spectra for TPU sample were fitted using three components. The first line centered at 285.0 eV indicates the presence of C-C type bonds, the second line at 286.3 eV indicates the presence of C-O-C groups, and the third line at 289.4 eV indicates the presence of N-C=O groups typical for polyurethane ^[1]^. The N 1s spectra were fitted with a single line centered at 400.1 eV, indicating the presence of N-C=O type groups typical for TPU polymer. The O 1s spectra were fitted with single line centered at 532.6 eV and indicate presence of both O-C and O=C type groups. The C 1s spectra for both PS and TPU-PS samples were similar and were fitted using three components. The first line centered at 284.1 eV indicates the presence of C=C type bonds as found for PS, the second line at 286.9 eV indicates the presence of C-O groups, and the third line at 291.0 eV comes from shake-up satellites typical for C=C type presence. The O 1s spectra for both samples were fitted with a single line centered at 531.9 eV, indicating the existence of O-C type bonds.

**Table S1.** Surface composition (atomic %) as determined by fitting XPS spectra.

| Sample | **C** | | | **N** | **O** |
| --- | --- | --- | --- | --- | --- |
|  | 285.0 | 286.3 | 289.4 | 400.1 | 532.6 |
|  | C-C | C-O-C | N-C=O | N-C=O | O-C  O=C |
| TPU | 45.3 | 33.4 | 1.2 | 2.8 | 17.4 |
|  | **C** | | | - | **O** |
|  | 284.1 | 286.9 | 291.0 | - | 531.9 |
|  | C=C | C-O | shake-up | - | O-C |
| PS | 91.6 | 3.0 | 3.4 | - | 2.0 |
| TPU-PS | 90.3 | 4.2 | 3.2 | - | 2.3 |

**
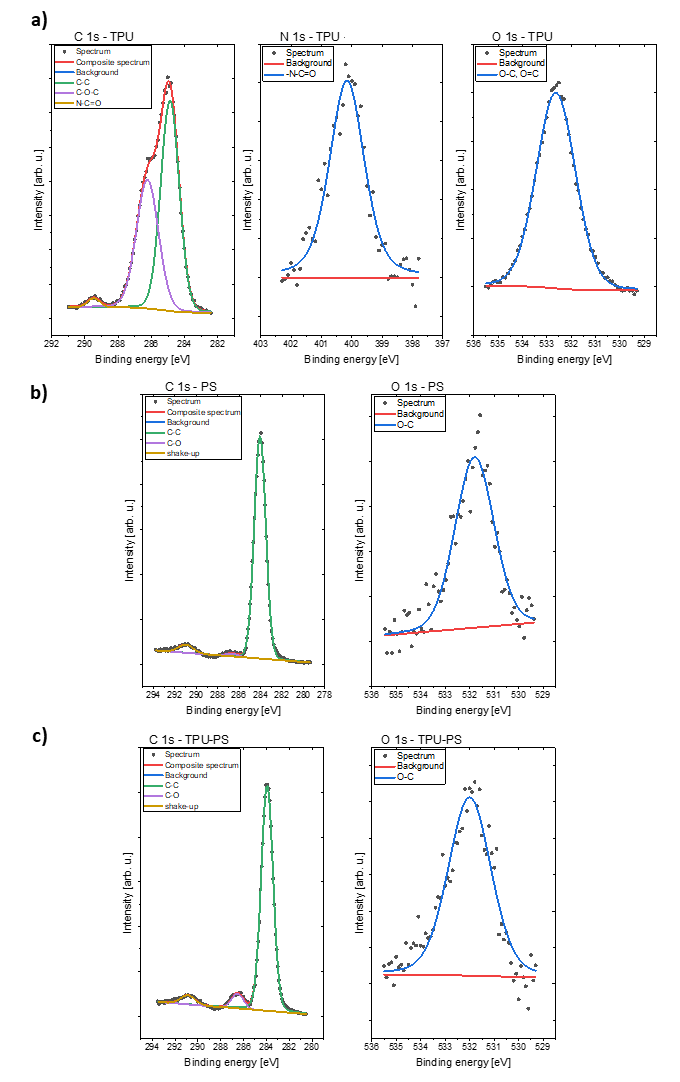
**

**Figure S4.** The comparison of XPS of: a) TPU, b) PS, c) TPU-PS.

Notable characteristic TPU peaks from stretching vibrations of the C-N were identified at wavenumbers of 1107, 1310, and 1414 cm^-1^, and bending vibrations of the N-H bands appeared in 1531 cm^-1^. Stretching vibrations of the C-O in the hard segment of TPU were detected at wavenumbers of 1069, and 1222 cm^-1^. However, it is also possible that the value of 1069 cm^-1^ is responsible for the in-plane bending of the C-H band in the PS phenyl ring. Moreover, the stretching vibrations of the C=O has appeared at 1701 cm^-1^, what is characteristic for the carbonyl group in TPU. Asymmetrical stretching vibrations of the CH_2_ bands were observed at 2921 cm^-1^, which are associated with both TPU and PS.

For PS, characteristic skeletal stretching vibrations of the C-C in the aromatic ring were identified at 1452, 1492, and 1600 cm^-1^. In-plane bending of the C-H in the PS phenyl ring was detected at 1028 cm^-1^, while aromatic stretching vibrations of the C-H were spotted at 3025 cm^-1^. Peaks, that showed out-of-plane bending bands of the C-H were noticed at 539, 696, and 754 cm^-1^. The comparison of FTIR spectra between electrospun fibers and pure granules did not indicate any structural changes resulting from the electrospinning, as shown in **Figure S4**.


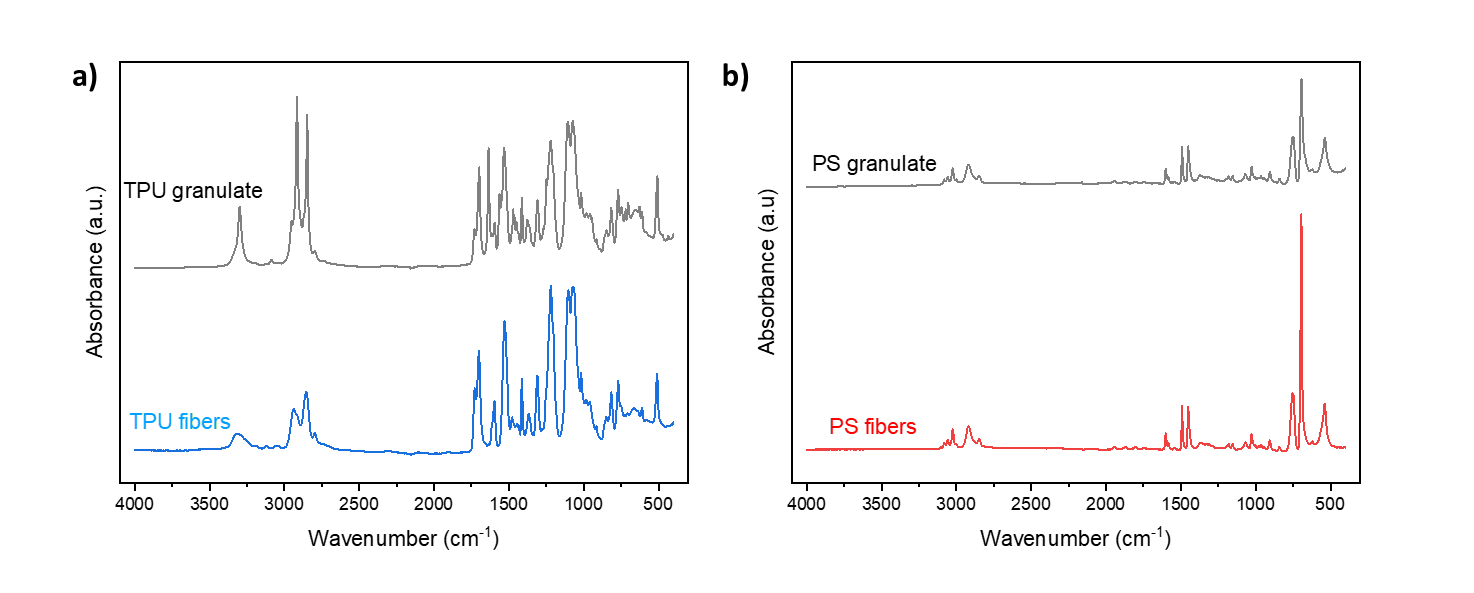


**Figure S5.** The comparison of FTIR-ATR spectra on pristine electrospun fibers and pure polymer granulate: a) TPU, b) PS.

**Table S2**. The FTIR analysis of TPU, PS and TPU-PS core-shell fibers.

| **Wavenumber (cm^-1^)** | | | **References**  ^[2–5]^ | **Functional groups** |
| --- | --- | --- | --- | --- |
| **TPU** | **PS** | **TPU-PS** |  |  |
| - | 539 | 539 | 540 | Out-of-plane bending bands of the C-H |
| - | 696 | 696 | 697 | Out-of-plane bending bands of the C-H |
| - | 754 | 754 | 757 | Out-of-plane bending bands of the C-H |
| - | 1028 | 1028 | 1028 | In-plane bending of the C-H in the PS phenyl ring |
| 1068 | 1069 | 1069 | 1073 (TPU)  1069 (PS) | Stretching of the C-O in hard segment of TPU;  In-plane bending of the C-H in the PS phenyl ring |
| 1103 | - | 1107 | - | Stretching vibrations of the C-N |
| 1220 | - | 1222 | 1219 | Stretching vibrations of the C-O |
| 1309 | - | 1310 | 1306 | Stretching vibrations of the C-N |
| 1413 | - | 1414 | 1412 | Symmetric stretching vibrations of the C-N |
| - | 1451 | 1452 | 1452 | Skeletal stretching vibrations of the C-C in the aromatic ring |
| - | 1492 | 1492 | 1493 | Skeletal stretching vibrations of the C-C in the aromatic ring |
| 1529 | - | 1531 | 1534 | Bending vibrations of the N-H |
| - | 1601 | 1600 | 1601 | Skeletal stretching vibrations of the C-C in the aromatic ring |
| 1701 | - | 1701 | 1700  1730 | Stretching vibrations of the C=O |
| 2938 | 2920 | 2921 | 2923 (PS)  2917 (TPU) | Asymmetrical stretching vibrations of the CH_2_ |
| - | 3025 | 3025 | 3026 | Aromatic stretching vibrations of the C-H |
| 3317 | - | - | 3420-3480 | Stretching vibrations of the -OH |

**
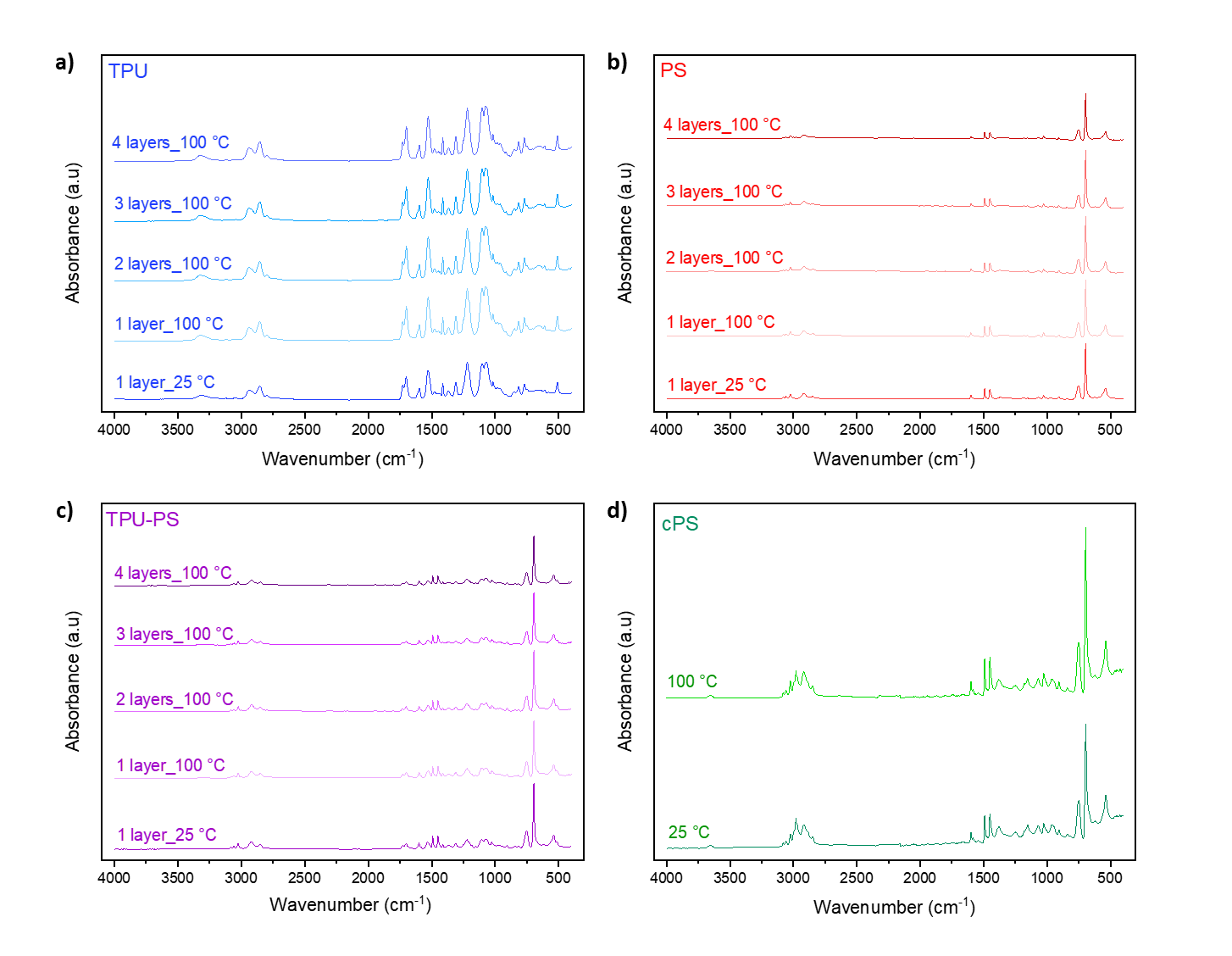
**

**Figure S6.** The comparison of FTIR-ATR spectra measured before and after heating at 100 °C for layering system of: a) TPU, b) PS, c) TPU-PS, d) commercial extruded polystyrene.

**Figure S7.** The DSC thermal curves for heating for TPU, PS, TPU-PS.


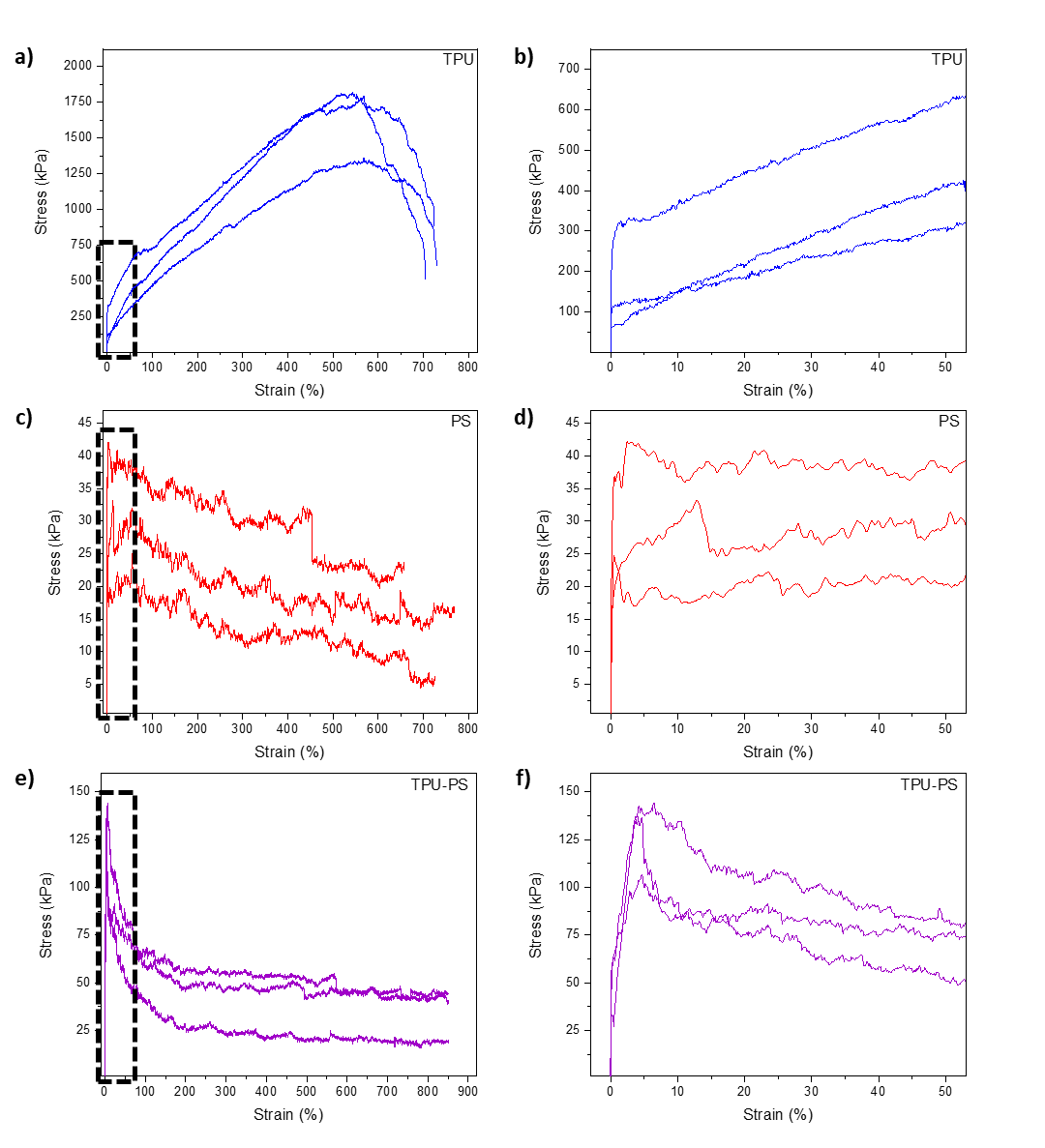


**Figure S8.** Stress – strain curves for membranes and their magnification in the deformation range for a-b) TPU, c-d) PS, and e-f) TPU-PS composite.


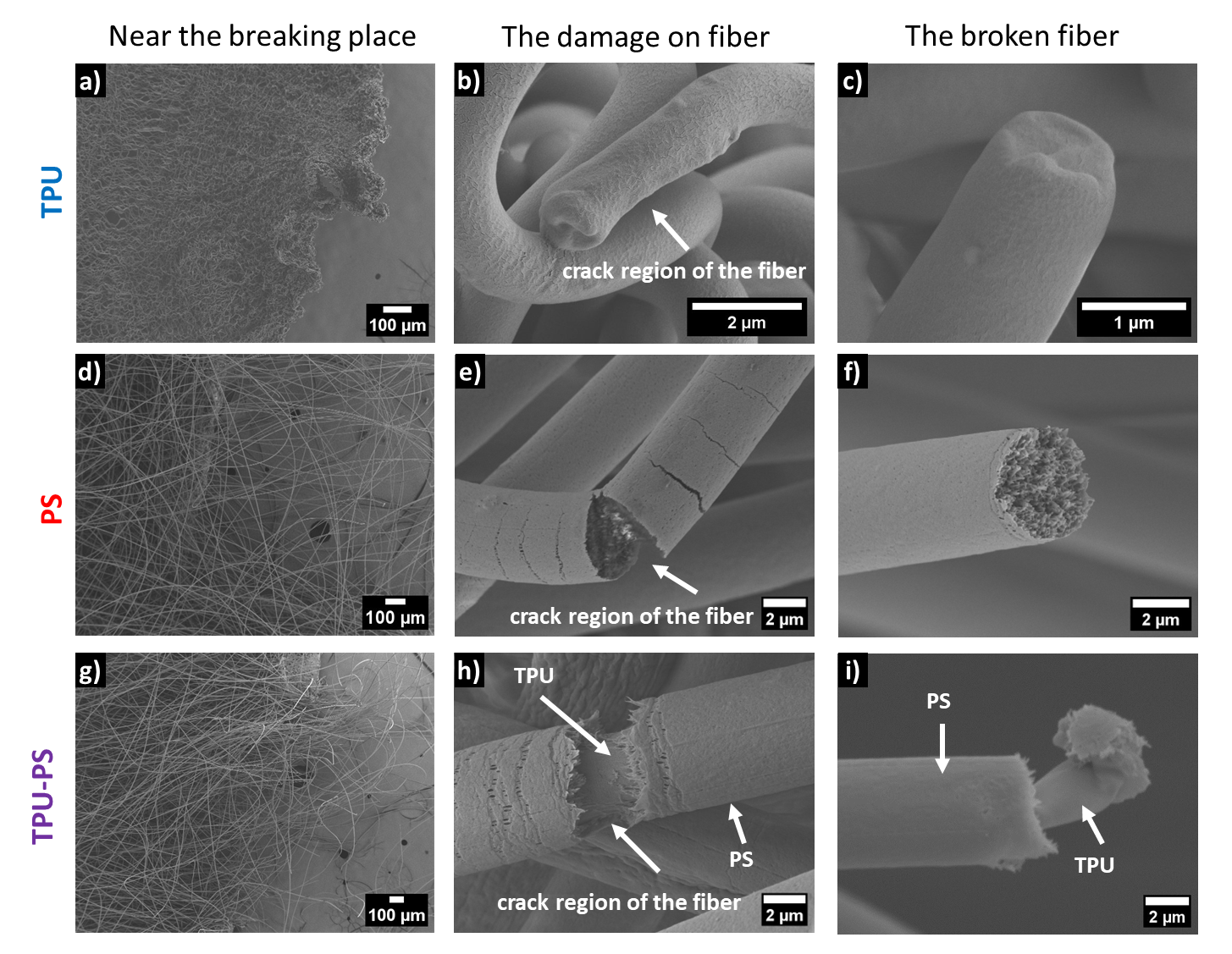


**Figure S9**. Images of fibers after mechanical testing: a-c) TPU, d-f) PS, and g-i) TPU-PS.


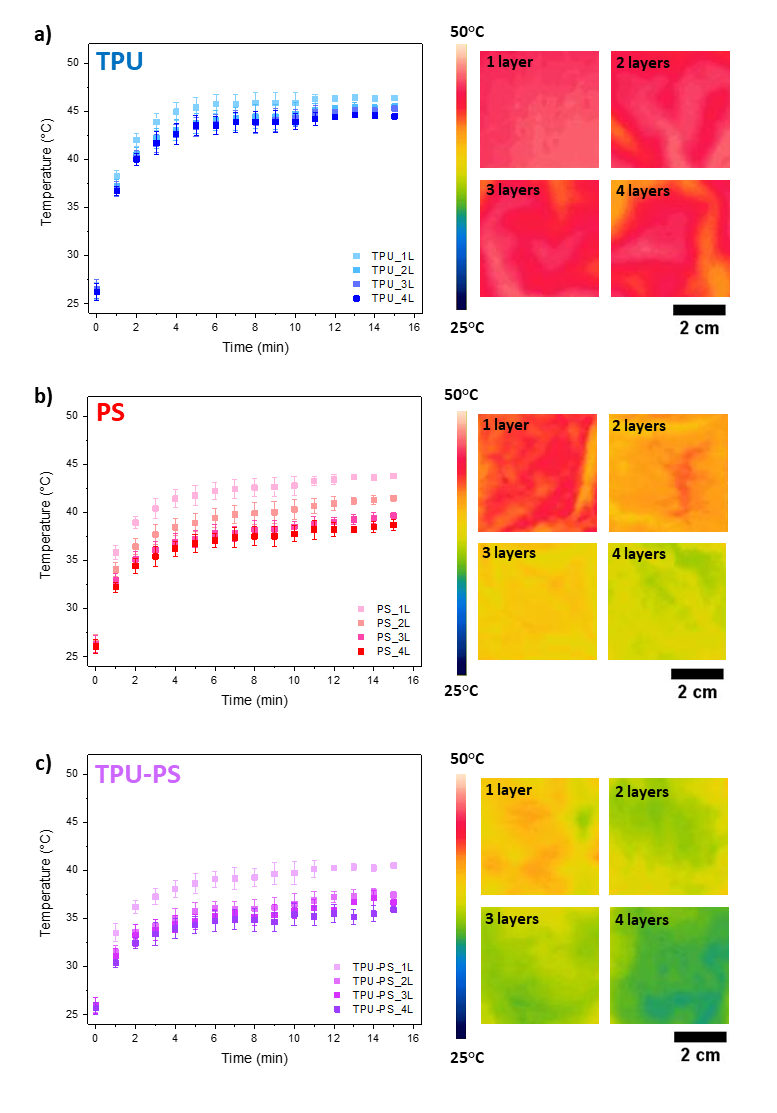


**Figure S10**. The heating curves for different number of layers and their thermal images after 15 min of experiment for: a) TPU, b) PS, c) TPU-PS.


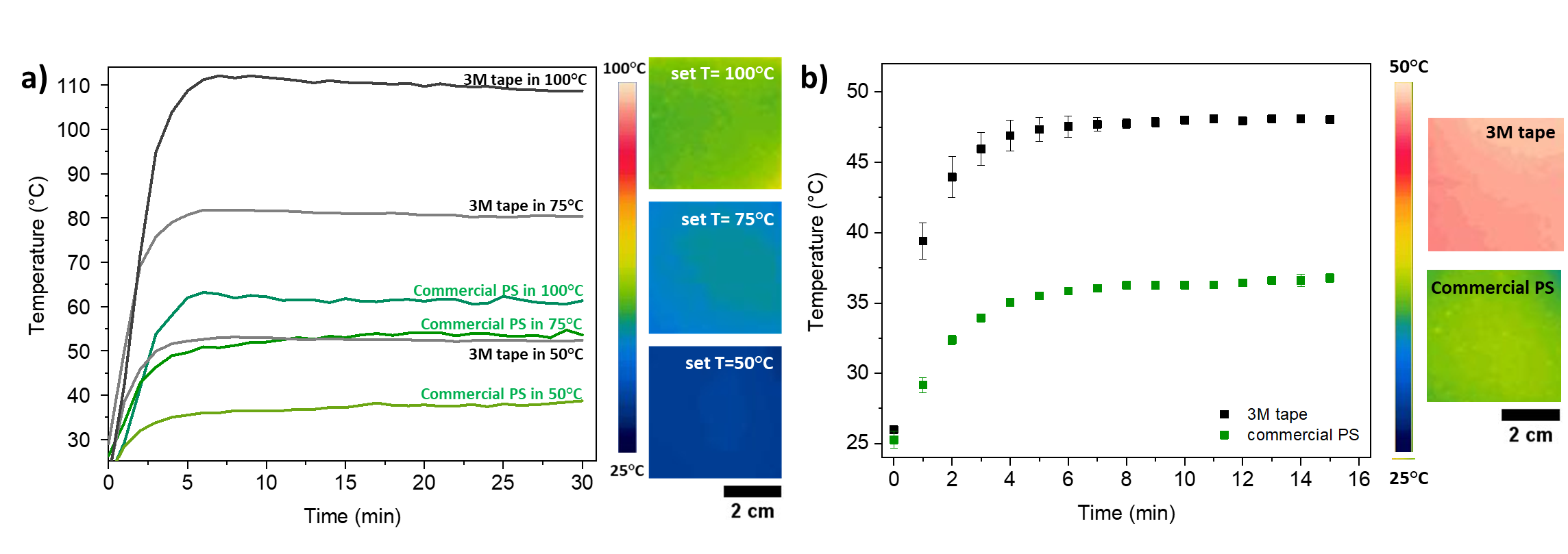


**Figure S11.** The results curves from the thermal test on the heating plate for a) the commercial extruded polystyrene and 3M tape at the set temperature of 50, 75 and 100 °C, b) and repeating measurements at a temperature of 50 °C. Thermal images presented in figure a) show the extruded polystyrene sample.


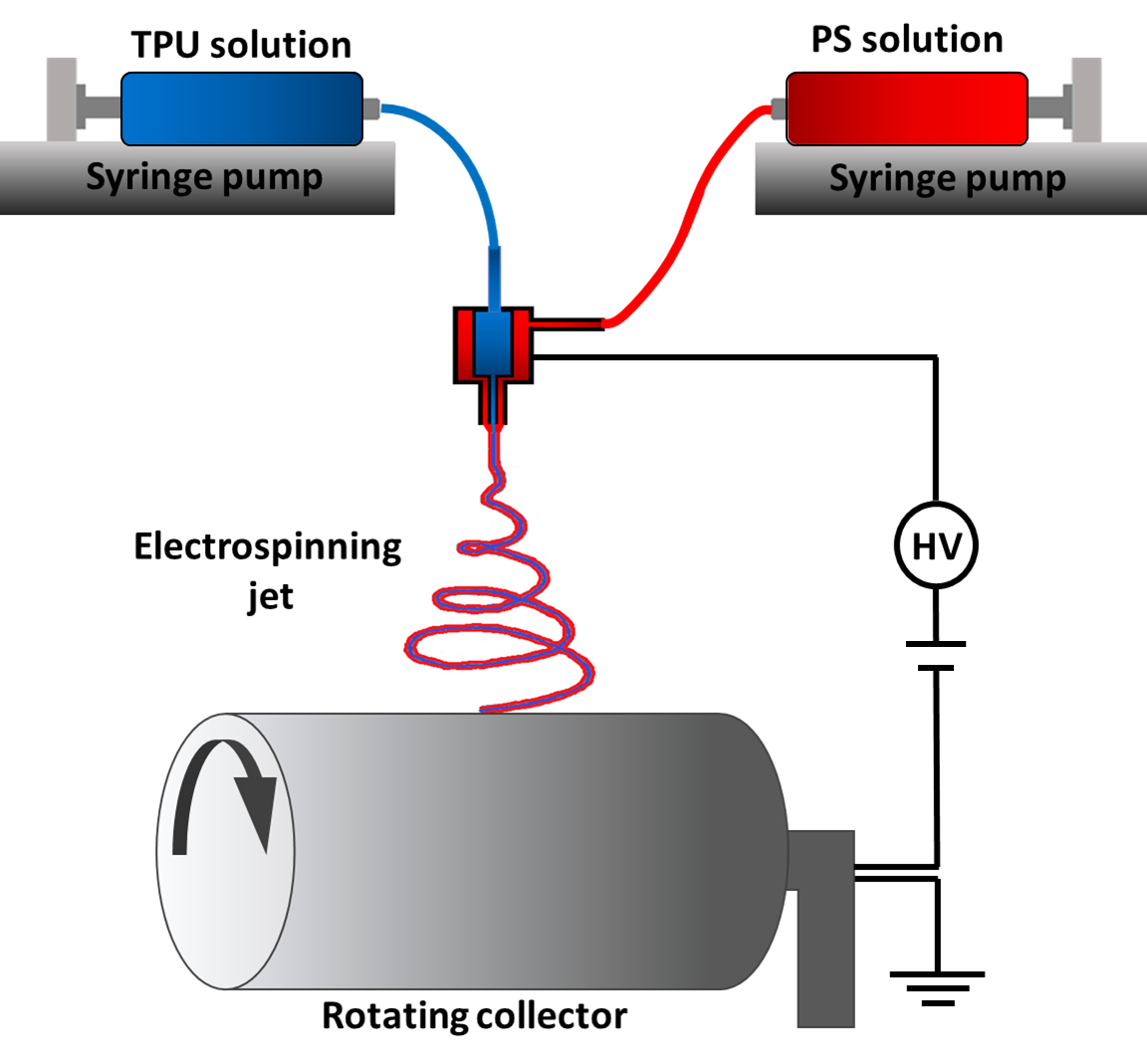


**Figure S12.** The setup for co-axial electrospinning.

**
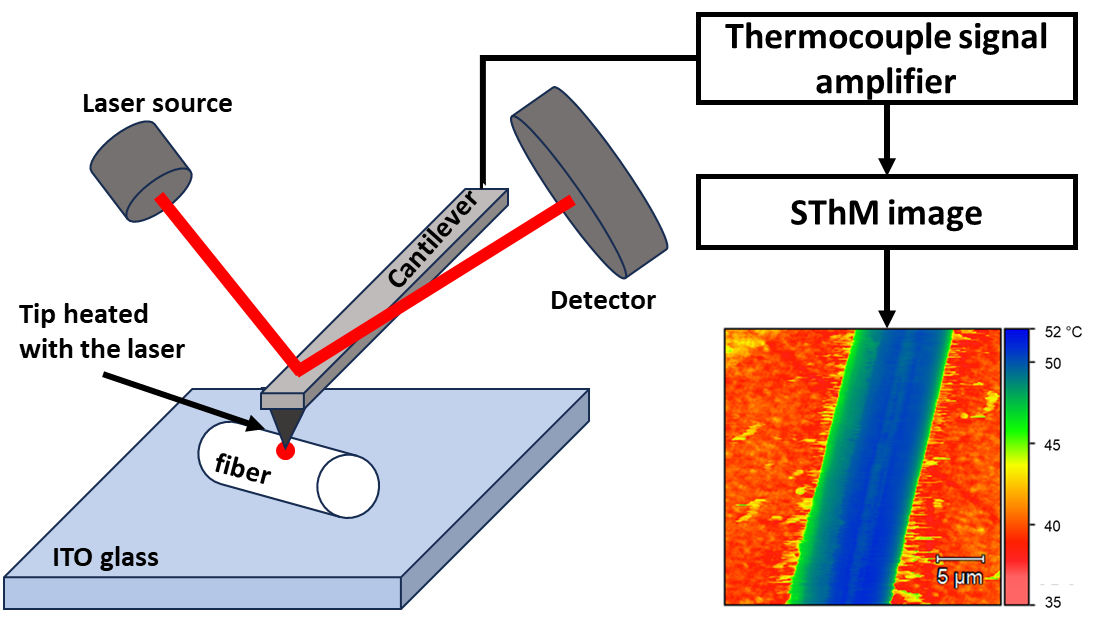
**

**Figure S13.** The methodology for conducting Scanning Thermal Microscopy measurements.

**
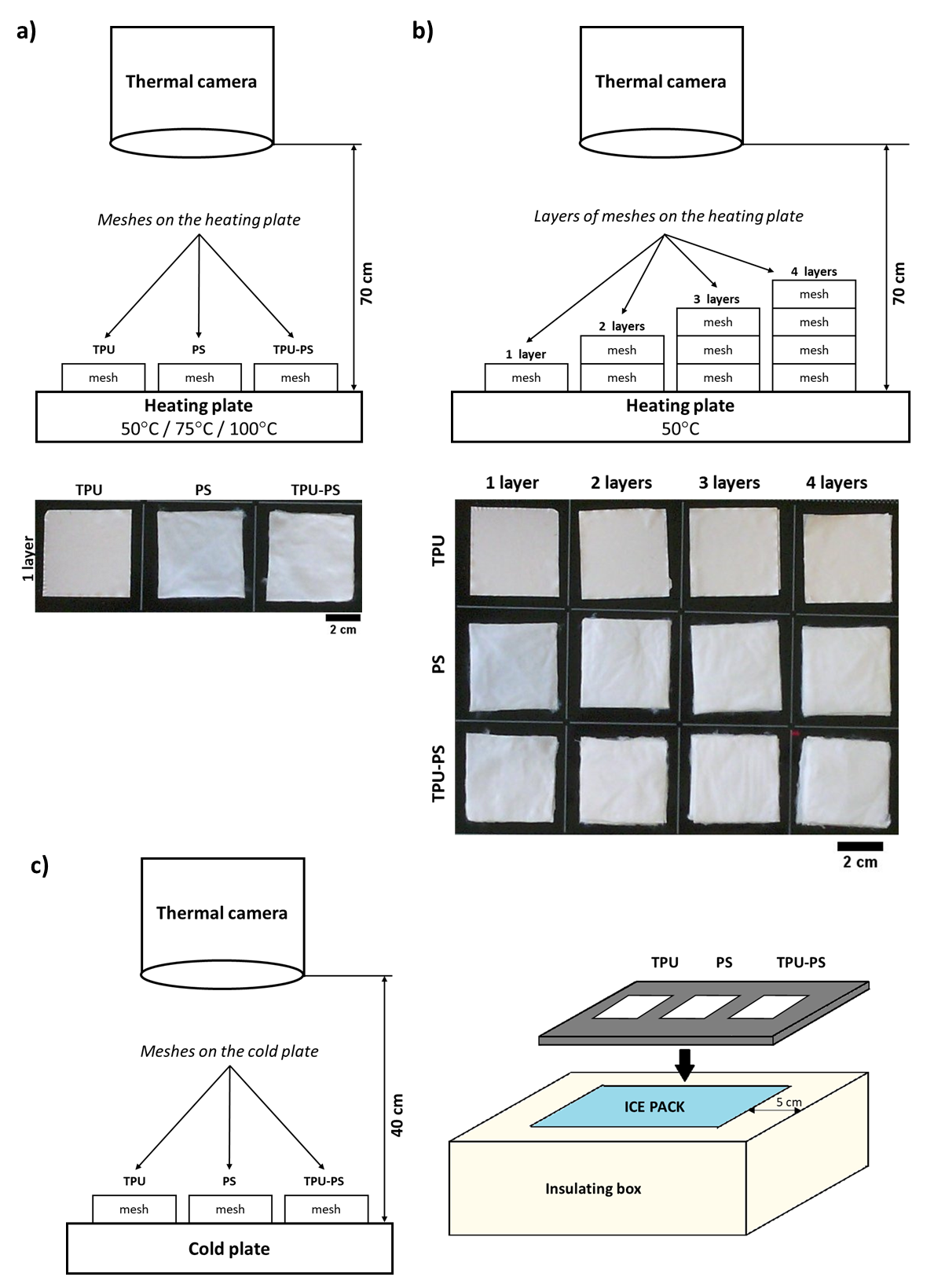
**

**Figure S14.** The schematic with a side view of the experimental setup designed for thermal camera measurements and the top view image of horizontally placed fiber samples on the heating plate for: a) a single electrospun layer and b) a different number of electrospun layers, and c) samples on the cold plate for a single electrospun layer. The mesh layers are not to scale for clarity.

**
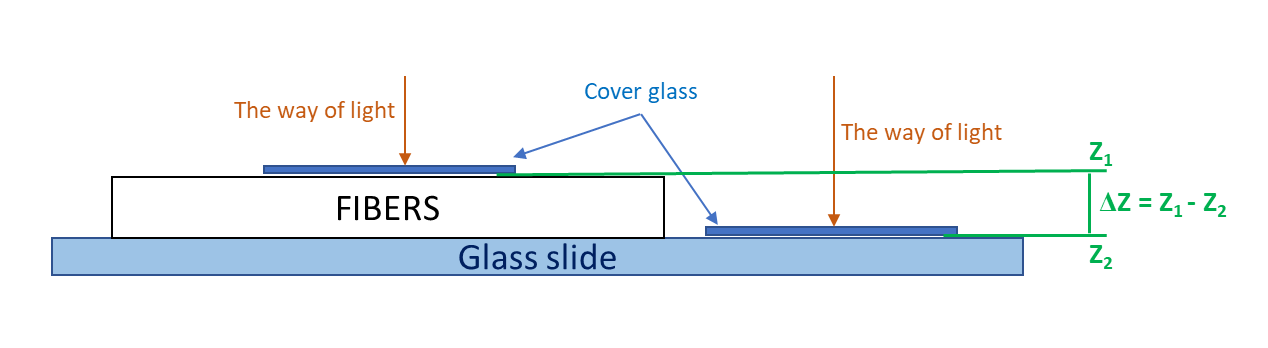
**

**Figure S15.** The scheme of the thickness measurement of fiber membranes and of the way of light.

**Table S3.** The thickness of prepared layer systems for thermal camera measurement.

| **Amount**  **of layers** | **TPU** | **PS** | **TPU-PS** |
| --- | --- | --- | --- |
|  | **[mm]** | | |
| **1** | 0.14 ± 0.01 | 1.33 ± 0.01 | 1.35 ± 0.06 |
| **2** | 0.18 ± 0.01 | 1.82 ± 0.09 | 2.15 ± 0.04 |
| **3** | 0.30 ± 0.02 | 2.11 ± 0.01 | 3.52 ± 0.01 |
| **4** | 0.42 ± 0.01 | 3.12 ± 0.06 | 4.44 ± 0.09 |

**Table S4.** The bulk density of fiber samples and commercial materials for thermal camera measurement.

| **TPU** | **PS** | **TPU-PS** | **Commercial extruded PS** | **3M tape** |
| --- | --- | --- | --- | --- |
| **[g∙cm^-3^]** | | | | |
| 0.105 ± 0.021 | 0.014 ± 0.003 | 0.016 ± 0.002 | 0.033 ± 0.001 | 1.425 ± 0.001 |

**
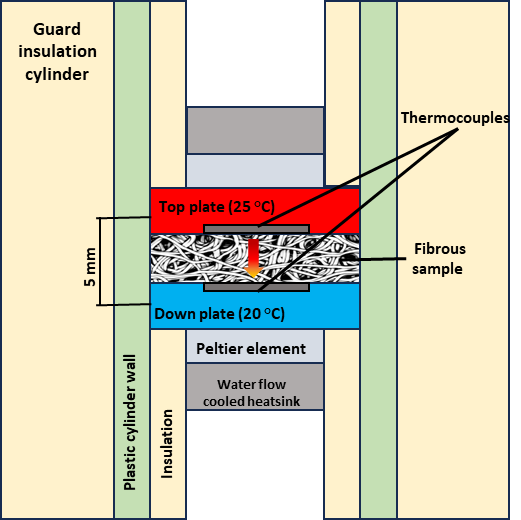
**

**Figure S16.** The schematic diagram for the thermal conductivity coefficient measurement.


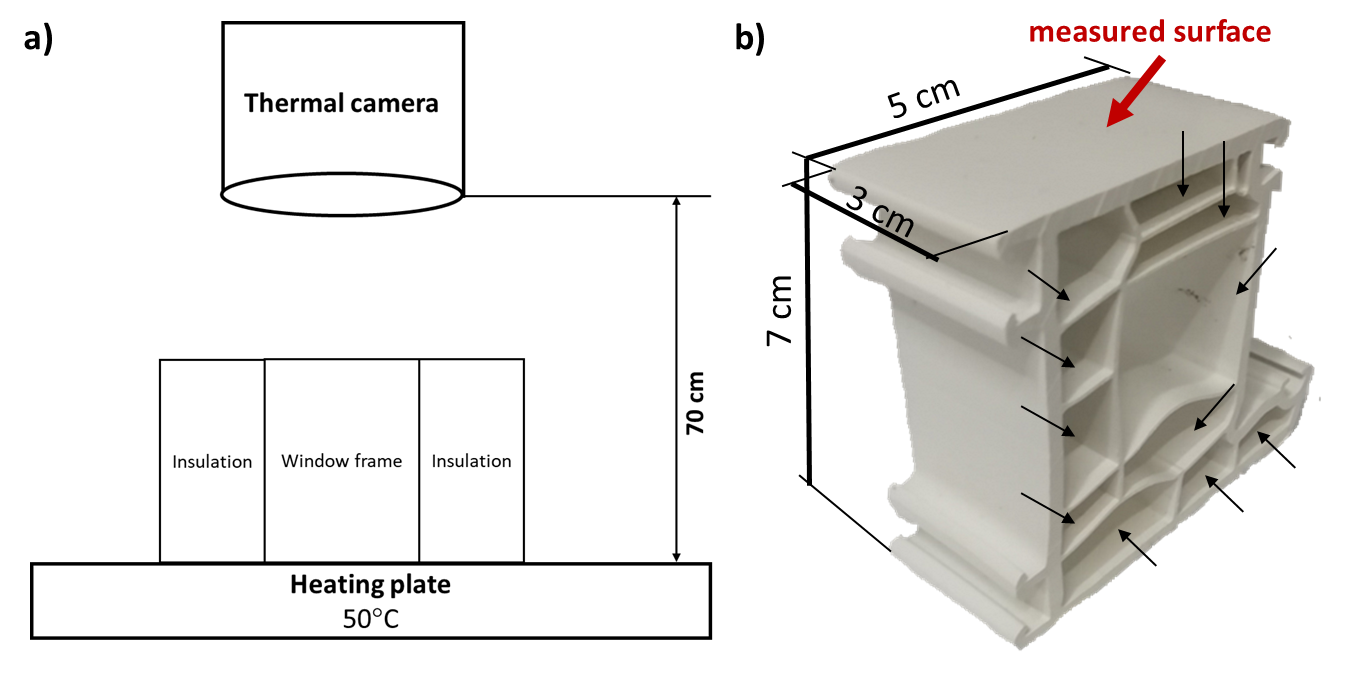


**Figure S17.** a) The schematic with a side view of the experimental setup designed for thermal camera measurements with window frame, and b) the empty measured part of window frame.

**REFERENCES**

[1] H. Bennett, G. J. Oliver, *J Chem Educ* **1993**, *70*, A25.

[2] D. Olmos, E. V. Martín, J. González-Benito, *Phys. Chem. Chem. Phys.* **2014**, *16*, 24339.

[3] E. Baştürk, S. Madakbaş, M. V. Kahraman, *Materials Research* **2016**, *19*, 434.

[4] C. H. Park, C. H. Kim, L. D. Tijing, D. H. Lee, M. H. Yu, H. R. Pant, Y. Kim, C. S. Kim, *Fibers and Polymers* **2012**, *13*, 339.

[5] S. Banikazemi, M. Rezaei, P. Rezaei, A. Babaie, A. Eyvazzadeh‐Kalajahi, *Polym Adv Technol* **2020**, *31*, 2199.
